# Increased Right Ventricular Repolarization Gradients Promote Arrhythmogenesis in a Murine Model of Brugada Syndrome

**DOI:** 10.1111/j.1540-8167.2010.01767.x

**Published:** 2010-10

**Authors:** Claire A Martin, Yanmin Zhang, Andrew A Grace, Christopher L-H Huang

**Affiliations:** * Physiological Laboratory, University of CambridgeDowning Site, Cambridge, United Kingdom; † Department of Paediatrics, First Affiliated Hospital, Xi'an Jiaotong UniversityXi'an, Peoples Republic of China; ‡ Department of Biochemistry, University of CambridgeDowning Site, Cambridge, United Kingdom

**Keywords:** arrhythmia, ion channels, sudden death, action potentials, conduction velocity, Brugada syndrome, sodium channels, ventricular tachycardia, antiarrhythmic drugs, repolarization

## Abstract

**Repolarization Gradients in Brugada Syndrome.***Introduction:* Brugada syndrome (BrS) is associated with loss of Na^+^ channel function and increased risks of a ventricular tachycardia exacerbated by flecainide but reduced by quinidine. Previous studies in nongenetic models have implicated both altered conduction times and repolarization gradients in this arrhythmogenicity. We compared activation latencies and spatial differences in action potential recovery between different ventricular regions in a murine *Scn5a*+/− BrS model, and investigated the effect of flecainide and quinidine upon these.

*Methods and Results:* Langendorff-perfused wild-type and *Scn5a*+/− hearts were subjected to regular pacing and a combination of programmed electrical stimulation techniques. Monophasic action potentials were recorded from the right (RV) and left ventricular (LV) epicardium and endocardium before and following flecainide (10 μM) or quinidine (5 μM) treatment, and activation latencies measured. Transmural repolarization gradients were then calculated from the difference between neighboring endocardial and epicardial action potential durations (APDs). *Scn5a*+/− hearts showed decreased RV epicardial APDs, accentuating RV, but not LV, transmural gradients. This correlated with increased arrhythmic tendencies compared with wild-type. Flecainide increased RV transmural gradients, while quinidine decreased them, in line with their respective pro- and antiarrhythmic effects. In contrast, *Scna5*+/− hearts showed slowed conduction times in both RV and LV, exacerbated not only by flecainide but also by quinidine, in contrast to their differing effects on arrhythmogenesis.

*Conclusion:* We use a murine genetic model of BrS to systematically analyze LV and RV action potential kinetics for the first time. This establishes a key role for accentuated transmural gradients, specifically in the RV, in its arrhythmogenicity. *(J Cardiovasc Electrophysiol, Vol. 21, pp. 1153-1159)*

## Introduction

Brugada syndrome (BrS) is associated with a loss of Na^+^ channel function and increased risks of polymorphic ventricular tachycardia (VT), potentially leading to sudden cardiac death. The arrhythmogenicity in BrS is thought to be a result of electrophysiological alterations localized to the right ventricle, reflected in ST elevation in the right precordial leads, right bundle branch block and changes specific to right ventricle (RV) epicardial action potential (AP) waveforms.^1^ Previous studies have implicated either slowed RV AP conduction^2,3^ or alterations in AP repolarization^1,4^ in the arrhythmogenesis associated with BrS. Thus, on one hand, reduced Na^+^ currents in BrS would be expected to compromise AP conduction. On the other, their reduction in relationship to *I*_to_ currents in BrS would shorten the RV epicardial APD and consequently permit reentrant waves.^5^

Experimental models for BrS have involved a number of pharmacological manipulations in canine systems. These have involved use of agents producing K^+^ channel opening by pinacidil, Na^+^ channel block by flecainide, Ca^2+^ channel block by verapamil, increased [Ca^2+^]_o_, metabolic inhibition or simulated ischemia. Such studies have implicated repolarization gradients caused by regional heterogeneities in electrical activity. However, there is uncertainty as to whether such pharmacological systems provide accurate or complete replication of the physiological changes underlying BrS. Furthermore, the pharmacological manipulations themselves inherently lack a specificity of action. Experimental systems using genetic modifications to replicate BrS may provide a more specific model to clarify physiological abnormalities associated with the disease condition.

Thus far, only the *Scn5a* gene, which encodes the cardiac Nav1.5 α-subunit, has been extensively studied in connection with BrS. Our previous studies using a heterozygotic *Scn5a*+/− mouse have recapitulated features of the human clinical condition in demonstrating an enhanced arrhythmogenesis that is exacerbated by flecainide and relieved by quinidine. This model has previously shown a 50% reduction in the transmembrane Na^+^ current and evidence for slowed ventricular and atrioventricular conduction.^6^ As yet, no evidence has been found for accentuated repolarization gradients in this model although to date no study of the RV has been made.^7^

In the present experiments, we seek for the first time in a genetic whole-heart BrS model to discriminate the contributions of alterations in conduction and repolarization gradients in the arrhythmogenesis, and to localize the pathophysiological mechanism in the RV. We were particularly interested in the effects upon conduction velocity and repolarization gradients of 2 clinically used drugs: flecainide, which is known to unmask ventricular arrhythmogenesis in otherwise asymptomatic BrS patients,^8^ and quinidine, which has shown protective actions in symptomatic BrS.^9^ We correlate alterations in RV gradients in the BrS model that are specifically exacerbated by flecainide, with the observed arrhythmogenic properties and with clinical findings implicating the RV in the arrhythmogenic mechanism. This may contribute to future work investigating possible pharmacological treatments for a disease for which the current mainstay of treatment is implantable cardioverter defibrillator (ICD) implantation.^5^

## Methods

### Langendorff Perfusion

Mice aged 4–8 months were obtained from breeding pairs of heterozygote *Scn5a*+/− and wild-type (WT) inbred 129/sv mice initially supplied by Harlan (UK). Experiments used a Langendorff-perfused preparation adapted for the murine heart, as described previously.^10^ Following the start of perfusion, viable hearts suitable for subsequent experimentation regained a pink coloration and spontaneous rhythmic contraction with warming. Where used, 10 μM flecainide and 5 μM quinidine (Sigma-Aldrich, Poole, UK) dissolved in buffer solution were perfused for 15 minutes prior to and throughout data acquisition. Concentrations were within the same range as known clinical therapeutic levels (flecainide: 0.2–0.9 mg L^−1^; quinidine: 2.0–5.0 mg L^−1^).^11^ All procedures conformed to the UK Animals (Scientific Procedures) Act 1986.

### Monophasic Action Potential Recording

Monophasic action potentials (MAPs) were recorded using an established contact-electrode technique detailed previously.^12^ Epicardial MAPs were recorded from the basal surface of the ventricular epicardium, both on the left and on the right using a miniaturized MAP electrode tip (Linton Instruments, Harvard Apparatus, UK). Endocardial MAPs used electrodes constructed from galvanically chlorided, Teflon-coated 0.25 mm diameter silver wire introduced into the ventricular cavity through a small access window created in the ventricular wall. Pilot experiments showed that both sets of electrodes provided equivalent MAP traces. MAP signals were amplified and band-pass filtered between 0.1 Hz and 300 Hz (Neurolog AC amplifiers and filters Models NL104 and NL125/6, respectively: Digitimer, Welwyn Garden City, Herts, UK), then digitized using a 1401plus interface (Cambridge Electronic Design, Cambridge, UK). Paired platinum stimulating electrodes paced the heart high on the interventricular septum.

The stimulating electrode and the epicardial LV and RV recording electrodes were clamped at a constant position through all experiments. This was at a distance of approximately 10 mm between stimulating and each recording electrode, although it was slightly larger for the LV than for the RV. This distance allowed the hearts to be placed into the rig, with the stimulating electrode coming into contact with the septum and the recording electrodes coming into contact with the left and right ventricles. While the direct absolute distance between electrodes would not necessarily reflect the path through which the electrical signal would be conducted in the spherical whole heart, the fact that the distance was maintained between experiments allowed consistent measurements of activation latencies. As the clamp was secure throughout experiments, this distance was constant to ∼0.5 mm, i.e., 5% of the distance between stimulating and recording electrodes.

For measurement of APDs, hearts were paced at 8 Hz for 5 minutes and recordings were made at LV and RV, epicardial and endocardial sites. For activation latencies, only epicardial sites were used, as the point of endocardial recording could not be accurately determined. For calculations of arrhythmia incidence, as well as regular 8 Hz pacing, the hearts also underwent an S1S2 protocol, imposing S2 extrastimuli following pacing S1 stimulus trains at S1–S2 intervals, reduced by 1 ms between successive drive trains until the preparation became refractory, and a dynamic pacing protocol progressively increasing pacing frequency every 100 beats. Thus for each heart, 12 recordings in total were made, with 3 protocols and 4 cardiac regions employed. MAP recordings were made both before and during treatment with either flecainide (10 μM) or quinidine (5 μM).

### Data Analysis

MAP waveforms were analyzed using Spike2 software (Cambridge Electronic Design). The point of maximum positive deflection was considered the point of 0% repolarization; that of full return to baseline of 100% repolarization. The intervening waveform was described in terms of APD_x_ measurements at x = 90% (APD_90_), 70% (APD_70_) and 50% (APD_50_) repolarization. The effect of heterogeneous repolarization on the difference between different cardiac regions was expressed empirically as the difference (ΔAPD_x_) between neighboring APD values: LV transmural = LV endocardial APD—LV epicardial APD, RV transmural = RV endocardial APD—RV epicardial APD, epicardial transventricular = LV epicardial APD—RV epicardial APD, endocardial transventricular = LV endocardial APD—RV endocardial APD.

Activation latencies for MAPs recorded from the RV and LV epicardium were measured from the stimulus time to peak amplitude of the MAP to give an indication of conduction velocities. With the positions of the stimulating and recording electrodes constant between experiments, comparisons could be made between WT and *Scn5a*+/− hearts, and before and after drug. However, as the distance from stimulating electrode on the septum was slightly greater to the LV recording electrode than the RV recording electrode, no direct comparison could be made between LV and RV. For the calculation of arrhythmic incidence, each run was labeled as either nonarrhythmic or showing one of 3 possible arrhythmias: VT (exceeding 1 second in duration), nonsustained VT (nsVT, lasting less than 1 second), or having one or more ventricular ectopic (VE).

### Statistical Procedures

Sixteen WT and 16 *Scn5a*+/− mice were used in our experiments, as this was a number sufficient to enable categorical statistical analysis for incidence of arrhythmogenesis. Although we also attempted quantitative measurements in all 16, we only included measurements from hearts whose MAPs strictly attained the accepted criteria of rapid upstroke phases, consistent amplitudes, smooth contoured repolarization phases and stable baselines^13^ in all 4 cardiac regions. We consequently used 12 hearts of each genotype for the calculation of activation latencies and repolarization gradients. In all cases, half of each group was exposed to flecainide and 6 to quinidine.

All results were expressed as mean ± S.E.M. values. The significance of differences in arrhythmic incidences used Fisher exact and Chi-squared tests. Differences in activation latencies, APDs and ΔAPDs between different regions were analyzed using Student's *t*-tests; these were paired where results could be compared from the same heart. Previously, ANOVA with post-hoc Tukey's honestly significant different tests have been used to analyze multiple paired data sets; however, this assumes that all data sets are compared with each other and thus increases the likelihood of Type II errors. We accordingly adopted a procedure using a modified Bonferroni correction factor^14^ using the following procedure: *t*-tests were grouped into independent data sets and the significance values rank-ordered from smallest to largest. The significance of the test with P-value at alpha/(number of tests) was evaluated and, if statistically significant, the test result from the test with the next smallest significance value was selected and evaluated at alpha/(number of tests—1), and so on. Alpha was set at 0.05.

## Results

### *Arrhythmias Were Unmasked by Flecainide in Scn5a*+/−*Mice, but Reduced by Quinidine*

**Figure 1:**
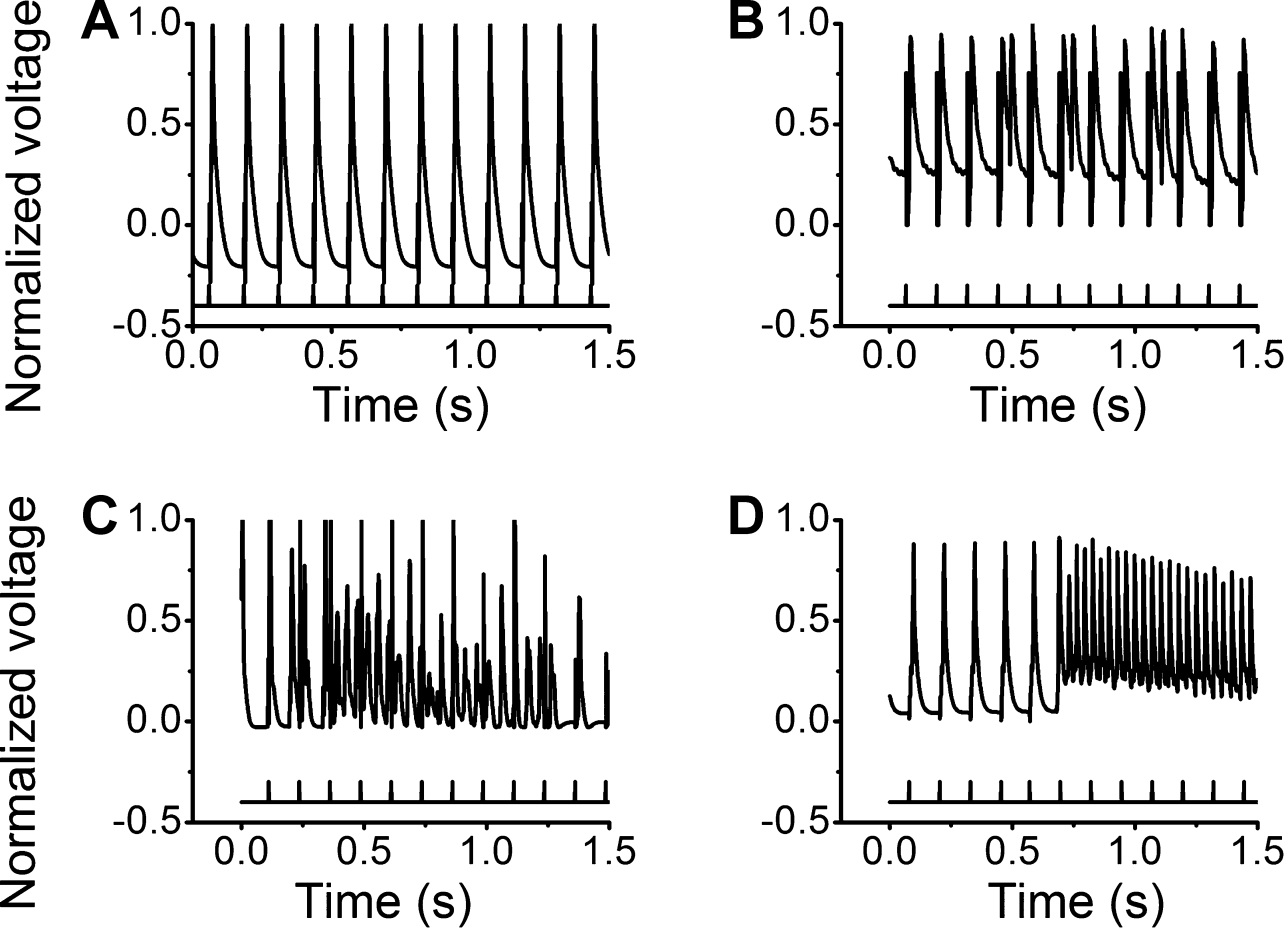
Representative RV epicardial MAP records from Scn5a+/−hearts during regular 8 Hz pacing. The vertical markers below each trace indicate stimulus timings. The y-axis is the MAP voltage normalized to peak MAP deflection. Hearts were either resistant to arrhythmia (A) or showed VEs (B), nsVT (lasting less than 1 second) (C), or VT (lasting more than 1 second) (D).

WT and *Scn5a*+/− hearts could either be resistant to arrhythmia or could show VEs, nsVT, or polymorphic VT during recording. Figure 1 illustrates these possible outcomes by showing RV epicardial MAP records from *Scn5a*+/− hearts during regular 8 Hz pacing. Similar traces could be obtained from WT, and from other cardiac regions in both *Scn5a*+/− and WT.

WT hearts showed low incidences of arrhythmia, even when subjected to PES protocols or with flecainide or quinidine treatment. In contrast, *Scn5a*+/− hearts showed markedly greater arrhythmic tendencies. In both cases, Fisher exact tests excluded significant differences in arrhythmia incidence between cardiac region or stimulus protocol (regular pacing, S1S2 or dynamic pacing protocols: see Methods). These results were therefore combined for Chi-squared testing sorted by genotype and drug condition (Table 1). WT hearts showed only small, insignificant increases in their low incidence of arrhythmogenesis with either drug treatment. However, flecainide and quinidine exerted contrasting effects in the *Scn5a*+/− hearts through all cardiac regions studied and protocols employed. *Scn5a*+/− hearts showed markedly increased arrhythmogenicity with flecainide, while quinidine reduced arrhythmic incidence in *Scn5a*+/− hearts to levels statistically indistinguishable from those normally observed in WT.

**TABLE 1:** The Incidence of Arrhythmogenesis in WT and *Scn5a*+/− Hearts Characterized as Showing One of Three Possible Arrhythmias: VT (Exceeding 1 second in Duration), nsVT (Lasting Less Than 1 second), and One or More VEs

	**WT**			***Scn5a*+/−**		
		
	**No Drug**	**Flecainide 10 μM**	**Quinidine 5 μM**	**No Drug**	**Flecainide 10 μM**	**Quinidine 5 μM**
VT	3/192	6/96	3/96	21/192	16/96	1/96
nsVT	5/192	2/96	4/96	8/192	12/96	1/96
VEs	2/192	0/96	1/96	3/192	9/96	1/96
Total	10/192 (5.2)	8/96 (8.3)	8/96 (8.3)	32/192 (16.7)*	37/96 (38.5)*^†^	3/96 (3.1)*^†^

Hearts showing more than one of these arrhythmias were marked as showing the more potentially clinically significant arrhythmic event, VEs being the least and VT being the most significant. Recordings were made from hearts either in the absence or presence of either flecainide (10 μM) or quinidine (5 μM). Results are expressed both as numbers of test runs showing arrhythmia out of the total, and as percentages in parentheses. Sixteen WT and 16 *Scn5a*+/− hearts were used before drug, and 8 of each were exposed to each drug treatment. Each heart was tested in 4 cardiac regions and with 3 different protocols, leading to 192 runs before drug for each genotype, and 96 each for each drug. The results of Chi-squared tests are shown, sorted by genotype (corrected P < 0.05 significance denoted by*) and drug condition (denoted by^†^).

### *Scn5a*+/−*Hearts Show Slowed Conduction, Which is Exacerbated by Both Flecainide and Quinidine*

**Figure 2:**
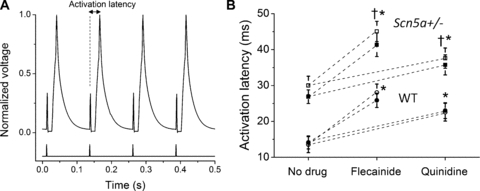
(A) MAP trace from a typical Scn5a+/−heart, showing measurement of activation latency from stimulus to peak amplitude of the MAP. (B) Activation latencies in the RV and LV epicardium from WT and Scn5a+/−hearts, before and after the addition of flecainide or quinidine. Open symbols denote LV; filled symbols denote RV. Twelve WT and 12 Scn5a+/−hearts were used, with 6 of each exposed to each drug. t-tests comparing values before and after drug have significant values (with P < 0.05) denoted by*. t-tests comparing WT and Scn5a+/−have significant values denoted by^†^. The same significant results occurred both in RV and LV, therefore only one set of symbols is shown.

In comparison with WT hearts, *Scn5a*+/− hearts showed longer activation latencies, corresponding to slowed conduction (Fig. 2). However, this effect was not localized to the RV epicardium, and was also true to the same extent in the LV. Both flecainide and quinidine increased activation latencies to similar extents (*t*-test: P = 0.09) in both the LV and RV of both WT and *Scn5a*+/− hearts.

### *Scn5a*+/−*and WT Hearts Show Regional Differences in AP Waveforms*

**Figure 3:**
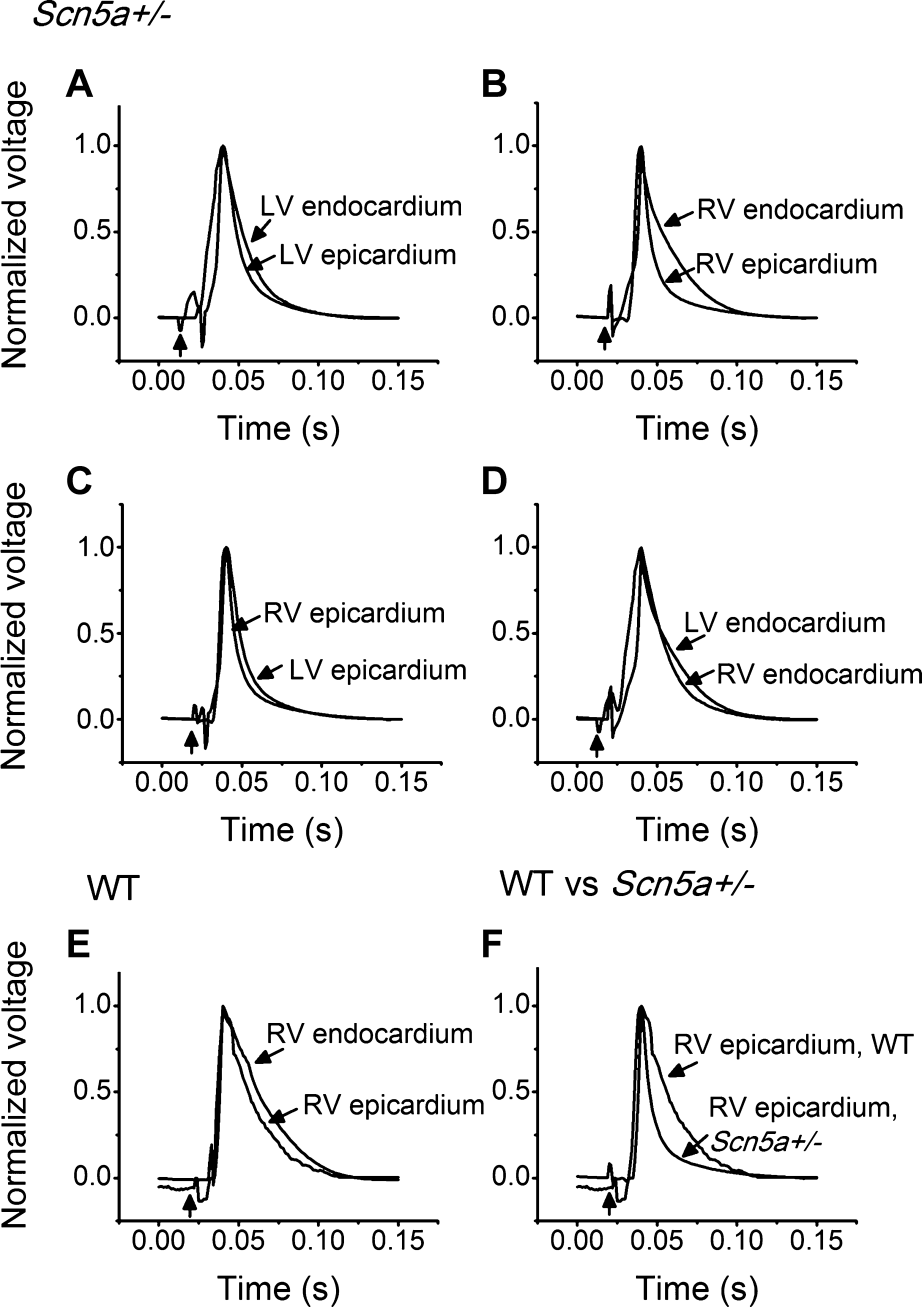
Typical MAP waveforms under 8 Hz pacing in (A)–(D) Scn5a+/−hearts, with traces overlaid to demonstrate the different resultant gradients: (A) LV epicardial and LV endocardial, (B) RV epicardial and RV endocardial, (C) LV and RV epicardial, (D) LV and RV endocardial. (E) RV epicardial and endocardial MAP waveforms in a typical WT heart and (F) RV epicardial MAPs compared between typical WT and Scn5a+/−hearts. The vertical arrow in each trace marks the pacing stimulus, which appears as an artifact on the trace.

**Figure 4:**
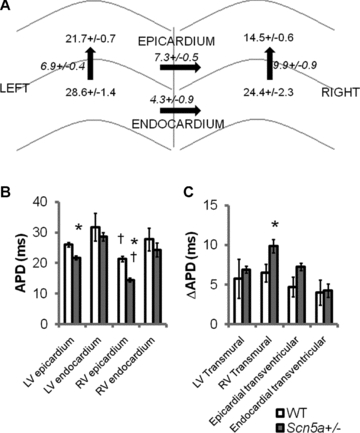
(A) Four chambers of a typical Scn5a+/−heart are displayed diagrammatically: left epicardium, right epicaridum, left endocardium, and right endocardium, with their respective APD_70_ values in normal font. Arrows between the 4 areas depict the gradients between them, with ΔAPD_70_ values in italic font. Data are expressed in ms as mean+/−SEM. Thus the gradients are calculated: LV transmural = LV endocardial APD—LV epicardial APD; RV transmural = RV endocardial APD—RV epicardial APD; epicardial transventricular = LV epicardial APD—RV epicardial APD; endocardial transventricular = LV endocardial APD—RV endocardial APD. (B) APD_70_ values obtained from the 4 ventricular regions and (C) gradients for WT and for Scn5a+/−hearts at the APD_70_ level. Twelve WT and 12 Scn5a+/−hearts were used. t-tests comparing APD_70_ values between cardiac regions within both WT and Scn5a+/−hearts have significant values (with P < 0.05) denoted by^†^. t-tests for both APD_70_ and ΔAPD_70_ values comparing WT and Scn5a+/−have significant values denoted by *.

We then undertook a systematic comparison of epicardial and endocardial MAP waveforms in both RV and LV. Figure 3A–D shows typical MAP waveforms in a typical *Scn5a*+/− heart, overlaid to demonstrate the contrasts in LV and RV transmural (Fig. 3A,B) and epicardial and endocardial transventricular gradients (Fig. 3C,D). Figure 3E shows typical RV epicardial and endocardial MAPs from a WT heart for comparison, while Figure 3F overlays WT and *Scn5a*+/− RV epicardial MAPs. Figure 4A shows the 4 areas of a typical *Scn5a*+/− heart, with APD_70_ values for each area shown in normal type. Arrows between the regions show the direction of each gradient, with its magnitude in italic type. Figure 4B shows APD_70_s for WT and *Scn5a*+/− hearts recorded from the 4 examined ventricular regions and (C) the resulting gradients between them expressed as ΔAPD_70_s across neighboring regions. Thus,

- Both untreated WT and *Scn5a*+/− hearts showed significant repolarization gradients varying between different cardiac regions. These gradients were particularly strong at earlier (i.e., 70% repolarization) phases of the AP recovery and arose from the following findings of the individual MAP durations. Endocardial MAP durations were very similar in the LV and RV. In contrast, epicardial MAP durations in the RV were shorter than those in the LV of both WT and *Scn5a*+/− hearts. Finally, both the RV and LV endocardial MAPs were longer than their corresponding epicardial MAPs, giving statistically significant differences in the RV in both WT and *Scn5a*+/− hearts. These differences resulted in RV transmural gradients that were significantly greater than the corresponding LV gradients, particularly in the *Scn5a*+/− hearts.
- *Scn5a*+/− hearts showed generally shorter APDs than WT. Although this was true of all ventricular regions, this only reached statistical significance in the RV epicardium. This resulted in significantly greater RV transmural gradients, as reflected in the ΔAPD_50_, ΔAPD_70_, and ΔAPD_90_ values in *Scn5a*+/− hearts compared with WT.

#### *Effects of Flecainide and Quinidine on AP Waveforms in Murine Scn5a*+/−*and WT Hearts*

The findings above implicate the RV in the arrhythmogenicity shown by *Scn5a*+/− through accentuated RV transmural repolarization gradients, brought about by shortened RV epicardial APDs. This hypothesis could be tested by assessing the extent to which the actions of flecainide and quinidine upon these gradients in *Scn5a*+/− compared with WT paralleled their respective pro- and antiarrhythmic actions in *Scn5a*+/−.

**Figure 5:**
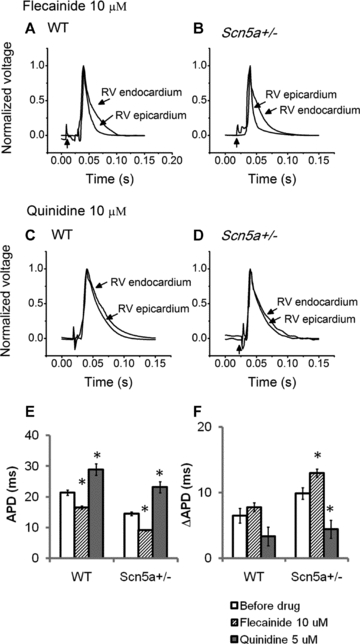
(A)–(D) MAP waveforms from RVs of (A), (C) WT and (B), (D) Scn5a+/−hearts subject to 8 Hz pacing following introduction of (A), (B) flecainide and (C), (D) quinidine. (E) APD_70_ measurements in the RV epicardium and (F) RV transmural gradients for WT and Scn5a+/−hearts before and following addition of either flecainide or quinidine. Six hearts were used in each group. t-tests comparing values before and after drug have significant values denoted by *.

The resulting experiments split the WT and *Scn5a*+/− hearts into age and sex matched groups for studies before and following treatment with 10 μM flecainide or 5 μM quinidine (n = 6 hearts in each group). Figure 5 shows MAP waveforms from RVs of the same WT and *Scn5a*+/− hearts as in Figure 3, now following introduction of (A, B) flecainide or (C, D) quinidine, overlaying epicardial and endocardial MAPs to illustrate how both drugs affect the RV transmural repolarization gradients. Then shown are (E) APD_70_s from the RV epicardium, and (F) the resultant RV transmural ΔAPD_70_s for WT and *Scn5a*+/− hearts before and after drug treatment.

These demonstrated the following:

- In WT hearts, flecainide shortened the APD values in all 4 ventricular regions investigated, but doing so significantly only for RV epicardium. This resulted in increases in the RV transmural gradients, but which only reached significance for the APD_90_.
- In WT hearts, quinidine increased the APD values in all the ventricular regions studied, but did so most noticeably in the RV epicardium, reaching significance here for all 3 repolarizations. The RV transmural gradient was reduced, but again only reaching significance for the APD_90_.
- In *Scn5a*+/− hearts, flecainide shortened the APD values in all the ventricular regions studied. Again, this effect was most noticeable in the RV epicardium. This resulted in marked increases in the RV transmural gradient whether measured as ΔAPD_50_, ΔAPD_70_, or ΔAPD_90_. There were no significant alterations in either the transventricular or the LV transmural gradient.
- In contrast, quinidine lengthened the APDs in all ventricular regions in *Scn5a*+/− hearts, again reaching significance specifically in the RV epicardium. These changes markedly decreased the RV transmural gradients to levels similar to that seen in WT before pharmacological treatment, but did not significantly reduce either the transventricular or LV transmural gradients.

The pharmacological maneuvers thus exerted similar patterns of effects upon *Scn5a*+/− as WT hearts, particularly involving the RV epicardium, with quinidine reducing, and flecainide increasing, the RV transmural gradients. However, the latter maneuvers acted upon a situation of already accentuated right transmural gradients in the *Scn5a*+/−, thereby giving very strongly positive RV transmural gradients.

## Discussion

The BrS has been associated with a heterogeneous group of genotypes; nevertheless, ∼15% of BrS patients do show a loss-of-function Na^+^ channel mutation. This prompted the recent development and use of a *Scn5a*+/− murine genetic model in studies of the BrS condition. Having first confirmed arrhythmogenicity in the *Scn5a*+/− hearts, we systematically investigated arrhythmic incidence in all 4 cardiac regions with 3 different stimulation protocols for the first time. We demonstrated that this model reproduces the clinical BrS condition by displaying ventricular arrhythmias that were exacerbated by flecainide, in line with its clinical effects in unmasking ventricular arrhythmogenesis in otherwise asymptomatic BrS patients,^8^ and reduced by quinidine, in line with its protective action on symptomatic BrS.^9^ Equal arrhythmia incidence in all 4 cardiac regions is consistent with arrhythmic activity arising in any one cardiac region ultimately spreading throughout both ventricles, therefore involving the entire heart. That stimulation protocol did not significantly affect arrhythmogenic incidence is consistent with clinical observations that the inducibility of VT during PES fails to predict sudden cardiac death in BrS patients.^15^

The differences in arrhythmic properties could be associated with both delayed epicardial activation latencies and increased right transventricular repolarization gradients in *Scn5a*+/− hearts. Such findings complement previous reports of *Scn5a*+/− myocytes having Na^+^ current amplitudes reduced to ∼50% of the levels shown by WT, as well as slowed AP conduction.^6^ Our study supports earlier work measuring regional APDs in canine^16^ and rat^17^ WT systems, as well as molecular and cellular studies of regional ion channel location.^18–21^ The present results also extend findings of altered repolarization gradients in canine wedge preparations that mimic BrS by pharmacological rather than genetic means.^22^ However, our study is the first using a genetic model for BrS and in which all 4 cardiac regions have been systematically compared.

Comparison of such altered conduction and repolarization properties through the epicardia and endocardia of the right and left ventricles demonstrated that while both slowed conduction and accentuated repolarization gradients could potentially contribute to the arrhythmogenic mechanism in BrS, it was only the repolarization abnormalities that could be localized to the RV epicardium. Thus, while conduction slowing may provide a background arrhythmic substrate, the RV origin of the arrhythmias in clinical BrS suggests that the heightened repolarization gradients in this region may be the specific trigger. These findings also shed light on the pathophysiology of ST elevation seen in BrS, for which 2 mechanisms have been proposed: either it is due to the difference in AP morphology between the RV epicardium and endocardium,^23^ or it is secondary to conduction slowing.^24^ The localization of the enhanced repolarization gradients to the RV may suggest that the physiological mechanism underlying ST elevation is also more closely related to a repolarization disorder than a delay in depolarization.

The increase in both latencies and transmural gradients produced by flecainide correlates with its proarrhythmic effects in both the model and clinical situations. The former finding parallels the clinical findings implicating slowed conduction in the RV outflow tract in Brugada patients^2^ that is exacerbated with class 1C drug challenge.^3^ The latter result supports findings in canine models, where high flecainide concentrations result in loss of the AP dome and marked AP abbreviation,^25^ and clinical studies demonstrating that pilsicainide administration in BrS patients triggers T wave alternans, which is known to reflect enhanced spatial and temporal dispersion of repolarization.^26^ However, whereas the action of flecainide on activation latencies occurred in both the RV and the LV, its action on transmural repolarization gradients was again significant specifically for the RV.

The increase in conduction delay produced by quinidine was not unexpected given its effect in blocking Na^+^ channels, but is in contrast to its effect in decreasing arrhythmic incidence in murine *Scn5a*+/− and human BrS hearts. On the other hand, its action in reducing transmural repolarization gradients correlated with its antiarrhythmic effects. Quinidine has correspondingly been shown to reverse the associated electrocardiographic abnormalities and prevent phase 2 reentry and polymorphic VT in experimental canine BrS models,^22^ and normalize the ST segment in clinical circumstances.^27,28^

The main limitations of the study are associated with the differences between mouse and human physiology. The small size of the mouse heart means that strong electrotonic forces may act to minimize the effect of any spatial heterogeneities that are created and reduce their potential to create reentrant substrate. The AP morphology differs between humans and mice, as mice use less L-type Ca channel current, which means that the mouse AP does not have a plateau phase and has a shorter APD.^29^ This means that the spike and dome morphology present in larger mammals is not present in our mouse model. However, despite these difficulties, we were able to demonstrate a clear propensity to arrhythmia in our BrS mouse model and correlate this with enhanced RV transmural gradients.

## Conclusion

In summary, this study has for the first time in a genetically modified BrS model assessed activation latency, APDs and derived AP repolarization gradients from 4 regions of the heart under a range of stimulation protocols and a range of pharmacological conditions. The experiments correlate the increased arrhythmogenicity in *Scn5a*+/− hearts with both increased activation latency and decreased RV epicardial APD and increased RV ΔAPDs. However, while conduction delay was not localized specifically to the RV and was increased by both flecainide and quinidine, the respective pro- and antiarrhythmic effects of flecainide and quinidine could be directly correlated with their actions in increasing and decreasing RV ΔAPDs. Thus, these findings, while not excluding a contribution of conduction delay, support most closely an arrhythmogenic trigger based on reentry from abnormal repolarization gradients between RV epicardial and endocardial sites.
